# Perinatal Programming of Childhood Asthma: Early Fetal Size, Growth Trajectory during Infancy, and Childhood Asthma Outcomes

**DOI:** 10.1155/2012/962923

**Published:** 2012-02-08

**Authors:** Steve Turner

**Affiliations:** Child Health, Royal Aberdeen Children's Hospital, University of Aberdeen, Foresterhill, Aberdeen AB25 2ZG, UK

## Abstract

The “fetal origins hypothesis” or concept of “developmental programming” suggests that faltering fetal growth and subsequent catch-up growth are implicated in the aetiology of cardiovascular disease. Associations between reduced birth weight, rapid postnatal weight gain, and asthma suggest that there are fetal origins to respiratory disease. The present paper first summarises the literature relating birth weight and post natal growth trajectories to asthma outcomes. Second, issues regarding the interpretation of antenatal fetal ultrasound measurements are discussed. Finally, recent reports linking antenatal measurement and growth trajectory to early childhood asthma outcomes are discussed. Understanding the nature and timing of factors which influence antenatal growth may give important insight into the antecedents of early-onset asthma with implications for interventions.

## 1. Introduction

As a rule, mammals either have a short gestation and are born in an immature condition or have a long gestation but are mature at term and can run or swim shortly after birth. One exception to this rule is *Homo sapiens* who has a relatively long gestation but is still immature at term. There is therefore a relatively large window of opportunity for both antenatal and early post natal exposures to positively or negatively affect human development. The respiratory system provides one example of human development spanning across antenatal and post natal periods since the airways are established before the pregnancy reaches midpoint but alveoli start to appear during the third trimester through to at least three years of age [1]. Not unsurprisingly therefore, antenatal and post natal exposures have been implicated in the causation of respiratory disease.

**Figure 1:**
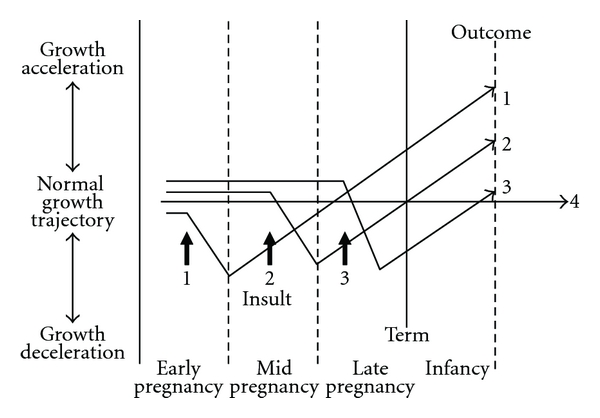
Schematic diagram demonstrating how growth deceleration at different gestations with resulting “catch-up growth” may result in low, normal, or high birth weight. Insult 1 in early pregnancy results in initial growth deceleration followed by growth acceleration during mid and late pregnancy and post natal life and is associated with increased birth weight (Outcome 1). Insult 2 occurs during mid pregnancy and results in growth deceleration followed by growth acceleration during later pregnancy and infancy, associated with normal birth weight (Outcome 2). Insult 3 occurs during late pregnancy leading to with low birth weight and ensuing “catch-up growth” during infancy (Outcome 3). Outcome 4 illustrates normal growth throughout pregnancy and infancy.

Describing the impact of antenatal and post natal exposures on development of the respiratory system is relatively easy after birth and methods include symptom-based questionnaires and physiological measurements. In contrast, determining the effect of antenatal exposures on the individual's respiratory wellbeing *in utero* is considerably more challenging. To date, birth weight has been commonly used as an index of fetal wellbeing and the assumption is that adverse exposures on the respiratory system are manifest as reduced growth. However, birth weight is the end point of nine month's growth and, as Figure 1 demonstrates, insults at different gestations might result in “catch-up growth” associated with high birth weight (outcome 1, Figure 1), normal birth weight (outcome 2, Figure 1), or low birth weight (outcome 3, Figure 1). For humans, the timing of a fetal insult and ensuing catch-up growth is not yet fully described, but the suggested growth trajectories depicted in Figure 1 can be inferred from animal studies [2] and also observational studies in humans [3, 4]. Growth acceleration may also (theoretically) be a primary phenomenon, that is, not a response to fetal growth failure. What is required is insight into fetal wellbeing during antenatal life and how this is relevant to post natal outcomes. Recently, longitudinal cohorts have been established which are able to link fetal ultrasound measurements made during pregnancy to post natal outcomes [4–9]. These studies are able to explore the fetal origins hypothesis, also termed developmental programming [10–12], which proposes that “fetal undernutrition in middle to late gestation, leads to disproportionate fetal growth, programs later (coronary heart) disease” [10]. The present review considers the relationship between fetal growth and childhood asthma, the latter being an example of a chronic condition where exposures in fetal life are thought to be important to aetiology. The review has three aims.

- To summarise recent evidence linking birth anthropometry and growth during infancy to respiratory outcomes.
- To explore the potential and limitations for using fetal ultrasound measurements as an index for fetal wellbeing.
- To describe the recent literature linking fetal size, fetal growth trajectories, and respiratory outcomes in later life.

## 2. Birth Anthropometry and Respiratory Outcomes

There is a considerable literature relating birth size to asthma symptoms in later life but no systematic review. There is a systematic review summarising the literature relating gestation to asthma [13] where the consensus is that a shorter gestation at delivery is associated with increased risk for the later development of asthma. For the purpose of the present article, key words “Asthma” and “Birth weight” were used in an Ovid search engine (to yield 129 abstracts) and relevant papers published since 2000 were reviewed. Publications known to the author but not identified by the search were added (see Table 1). A formal systematic review on this topic is required.

A number of themes emerge from this semistructured literature review. First, there is no consensus on the relationship between birth weight and asthma; nine studies find an inverse relationship [14–22], ten find no relationship [23–32], and three find a positive relationship [33–35]. There may be an issue of power since inverse relationships are seen in large and very large study populations (median of 8071 individuals) whereas studies reporting no effect tend to be of medium size (median number of participants 3628). Second, there is more consistent evidence of increased asthma among those of low birth weight (i.e., less than 2.5 kg) where there is approximately a doubling in risk for asthma; it is not clear whether this is independent of prematurity. Third, there is limited evidence of increased asthma among very heavy infants (i.e., >4.5 kg) [33, 34]. Finally, other indices of size at birth, for example, ponderal index [30, 35] (weight/length^3^), may be more closely related to asthma compared to birth weight.

Given the different growth trajectories which may converge to a given birth weight (Figure 1) it is perhaps not surprising that the relationship between birth weight and asthma is not straight-forward. Using birth weight as a “snap shot” of fetal wellbeing has its limitations and in recognition of this, post natal growth trajectory has been related to asthma.

## 3. Post Natal Weight Gain, Asthma, and Lung Function

Post natal growth trajectory may be a more accurate reflection of antenatal growth compared to birth weight; a movie picture rather than a photograph telling a story. For example, an infant born on the 9th centile for birth weight who demonstrates accelerated post natal growth and reaches the 50th centile by three months might be assumed to have had growth suppression *in utero*. In contrast, a similar-sized infant born on the 9th centile whose post natal growth follows the 9th centile is simply marginally smaller than average and most likely grew along the 9th centile *in utero*. Ideally antenatal and post natal growth trajectories would be obtained to study the relationship between early growth and asthma outcomes.

What is important when understanding the relevance of the relationship between somatic and pulmonary growth is that whilst the body may be able to “catch up,” the airways are established by mid pregnancy and may not be able to catch up, leaving the individual with small airways relative to body size. Proof-of-concept for dysanapsis (i.e., dissociation between somatic and pulmonary growth) comes from a cohort study of 1232 individuals from Chile study where increased gain in weight and length during infancy were associated with a modest increase in asthma symptoms at age 23–29 [36]. Studies where followup is only reported into childhood also find positive associations between weight gain during infancy and asthma risk. A cohort in Southampton reported that increased weight and adiposity (but not length) during infancy was associated with increased risk for wheeze at of age three years [7]. Paul and colleagues [37] used data collected as part of a randomised controlled trial of inhaled corticosteroids in wheezy 2-3-year-old children and related change in weight between birth and enrolment to asthma outcomes including burden of symptoms, quality of life, functional capacity, exacerbations of asthma and medication side effects. Compared with reduced growth, accelerated growth was associated with a 50% increase in exacerbations requiring prednisolone treatment (0.6/year/child versus 0.9/year/child) and more than a 100% increase in unscheduled physician visits (0.5/year/child versus 1.1/year/child). Although there is a relative paucity of data linking growth in infancy to asthma diagnosis and symptoms, this limited literature is entirely consistent. The literature linking weight gain and pulmonary function is less consistent.

In infancy, rapid early weight gain is associated with reduced FEV_0.4_ at one month [38] and reduced mid expiratory flow at the end of infancy [39] but a trend for increased mid expiratory flow at age 11 years [39]. In a large cohort study in the Avon region of UK, infants with birth weight < 10th centile who demonstrate catch-up growth had evidence of better lung function as 7-8 year olds compared to those of a similar weight who do not catch up but still lower than peers of average birth weight [40]. The improved lung function in the “catch-up” group was not significantly better than the persistently small infants despite this being a very large study population and the results could be interpreted as supporting the concept of dysanapsis or possibly indicating that catch up in somatic growth may be associated with very small degree of catch up in pulmonary growth [40].

Cohorts with followup into adulthood have demonstrated increased weight gain during infancy being associated with increased lung function in adults, that is, a reversal of the relationship seen between weight gain and lung function in infancy. For men aged 31 years, this was equivalent to a mean increase of 51 mLs FVC for each kg gained and for women, mean increases of 19 mLs FEV1 and 30 mLs FVC [41]. In a second study increased growth during first three years was associated with increased functional residual capacity and gas transfer at 32 years of age [42] but not with altered FEV_1_. The transformation of the association between post natal growth and lung function from negative in infants to positive in adults is difficult to understand but might be a cohort effect, that is, the nature of the relationship has changed over time.

Findings from these epidemiological studies could be interpreted as follows.

- A more rapid increase in size during infancy is associated with increased risk for asthma.
- A more rapid increase in size during infancy is associated with reduced lung function during infancy but marginally increased lung function during adulthood.

## 4. Application of Fetal Measurements to Epidemiological Studies

Ultrasonography provides a unique view of the developing fetus, and in many countries ultrasound examinations are routinely carried out to date pregnancies in the first trimester (usually at approximately week 10) and detect congenital abnormalities during and after the second trimester (approximately week 20). The interpretation of fetal measurements in the context of post natal outcomes is not necessarily straightforward and there are a number of methodological considerations each of which apparently weaken any relationship between fetal size and later health outcomes.

**Figure 2:**
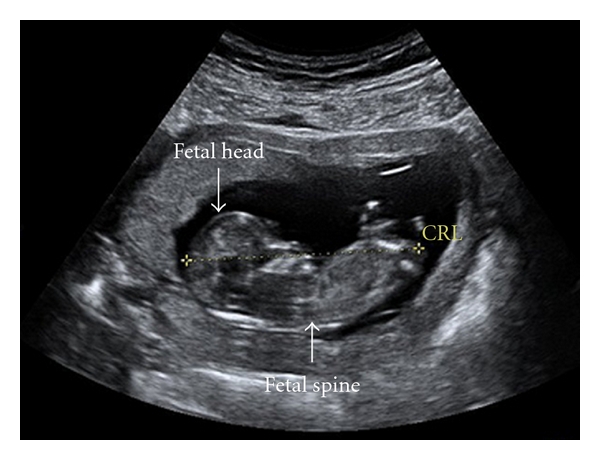
Ultrasound image of a 12-week fetus. The broken yellow line is the crown rump length (CRL) measurement.

First, different measurements are made at different gestations, so which measurement is “best”? Crown rump length (Figure 2) is measured in the first trimester to date a pregnancy and is known to be a very accurate predictor of gestation in the first trimester [43] but becomes more variable beyond 14 weeks [43] and other measurements are required. Second trimester fetal measurements include biparietal diameter [44], femur length [45], and abdominal girth [46] and are not routinely measured in the first trimester since the fetus is so small. Even modern ultrasound may lack precision for head, abdominal, and limb measurements in a ten-week fetus which on average measures 45 mm from crown to rump. At birth, weight, crown-heel length, and occipitofrontal circumference can be measured but these are not directly comparable to first and second trimester measurements. Unfortunately, there is no single “gold standard” fetal measurement which can be made throughout pregnancy. Second, even in health, fetal growth is not linear over time [47] and an apparent acceleration or deceleration may be normal. Third, in the context of fetal “stress” there is sparing of head growth at the expense of the body which leads to asymmetrical growth retardation; in the context of fetal “stress,” the fetal head measurement may be within the normal range but the abdominal girth will be reduced. Fourth, as with all measurements there is an element of interobserver variability and first trimester fetal measurements, this is estimated to be approximately 10% [48]. In epidemiological studies, the impact of factors such as nonlinear growth and intrasubject variation in measurements can be minimised by inclusion of large numbers of participants.

Finally, and perhaps most importantly, anthropometric measurements are not necessarily related to the function of individual organ systems, for example, cardiovascular system. However, spirometry is positively correlated with anthropometric measurements in children including sitting height [49] (i.e., crown rump length) and limb length [50] (i.e., femur length) and therefore it is biologically plausible that fetal measurements are a valid index of respiratory function.

## 5. Fetal Growth Trajectory and Asthma and Allergy Outcomes

At the time of writing, fetal growth has been related to childhood asthma outcomes in three reports from two cohorts [7, 9, 51]. Methodological differences between the two cohorts make direct comparison difficult but there are some patterns which emerge (Tables 2 and 3).

In a cohort recruited in Southampton [7], prospective fetal measurements were made at 10, 19, and 34 weeks gestation. The same fetal measurements were made on each assessment, that is abdominal and head circumference, and this allows relative change in the same measurement to be studied. The Southampton group have focussed on relative change in growth and have not yet reported associations between absolute fetal size and childhood asthma outcomes. In their paper, Pike et al. [7] report an association between reduced head circumference growth between weeks 10 and 19 and reduced growth in abdominal girth between weeks 19 and 34 and increased wheeze at age three. Contrasting associations between fetal growth trajectory and atopy at age three years were seen; increasing abdominal growth between 10 and 19 weeks but reduced growth of the same parameter between weeks 19 and 34 were linked to increased risk for atopy. Although based on a very young group of individuals, these findings provide proof-of-concept that factors which influence antenatal growth may be important to asthma and atopy. Additionally these findings may explain the inconsistent association between asthma and atopy, for example, increased growth in early pregnancy may increase atopy but reduce asthma risk whereas faltering growth in later pregnancy may be associated with both.

A second cohort, recruited in Aberdeen [9, 51], was primarily designed to relate dietary exposures to childhood asthma outcomes and fetal measurements from routine first trimester “dating” and second trimester “fetal anomaly” ultrasound examinations were retrieved retrospectively. This meant that not all fetal measurements could be retrieved and different fetal measurements were made in the first and second trimester, whilst third trimester measurements were those made at delivery, that is, birth weight, length, and head circumference. Strengths of the Aberdeen cohort include relatively extended followup at ages five and ten years, inclusion of physiological measurements in childhood (e.g., spirometry), and relation of absolute fetal size to asthma outcomes. The Aberdeen group have also looked at some maternal factors which affect fetal growth [8, 51].

At five years of age, the main message from the Aberdeen cohort was that reduced fetal size in the first trimester was associated with increased risk for asthma symptoms and obstructed lung function regardless of later fetal growth [51]. Those who were initially short and then became larger had worse asthma outcome compared to those who were persistently large. To place fetal measurements into context, the average fetus measured 46 mm at ten weeks and asthma risk at five years fell by an average of 5% for each mm increase in size.

As part of the evaluation of the Aberdeen cohort at age five years, fetal size was related to maternal diet and smoking during pregnancy. Reduced first trimester fetal size was associated with reduced maternal plasma alpha tocopherol (vitamin E) suggesting that maternal diet may be important to early asthma causation [51]. The concept that vitamin E may enhance fetal lung growth is supported by work in animal models [52, 53] but it is also possible that increased vitamin E is merely an index of a generally healthier maternal diet during pregnancy and a single nutrient is not likely to have a considerable impact on fetal growth in isolation. Additional factors associated with fetal growth were male gender which was associated with increased growth during first and second trimesters [51] and maternal smoking, which was associated with reduced femur length [8]. There are a number of mechanisms whereby maternal smoking may affect fetal and lung development. Carbon monoxide, a by-product of tobacco smoking, induces fetal hypoxia [54] which may directly induce fetal growth failure. Products of tobacco smoke can indirectly affect fetal growth via a negative influence on placental function [55]; for example, nicotine causes vasoconstriction in placental vessels [55]. Maternal smoking may also reduce fetal growth by suppression of placental growth hormone and fetal insulin-like growth factor endocrine function [56]. Finally, and not to the exclusion of the previous mechanisms, maternal (and also perhaps grandmaternal smoking) may induce epigenetic in the developing fetus which could increase the unborn child's risk for asthma in later life [57].

At ten years of age, the main message from the Aberdeen cohort remained that reduced first trimester fetal size was linked to a poorer asthma outcome. Asthma outcomes were worst in those who were persistently small, best in those who were persistently large, and intermediate for those with changing growth trajectories. Additionally, the investigators demonstrated that reduced first trimester fetal size was associated with asthma which was present at both ages five and ten years but not transient or later onset asthma symptoms. Birth weight and first trimester fetal size were independently associated with reduced lung function at ten years suggesting that an element of remodelling of the respiratory system may be taking place throughout pregnancy. At ten years, but not at age five years, there was evidence that increased early growth was associated with hayfever and reduced early growth was apparently protective for eczema; there was no association between changing growth trajectory and skin prick positivity at ten years.

Putting the results of the two cohorts together is not straightforward due to the differences in methodology, analytical approach and age at follow up, however some broad conclusions can be drawn. First, changes in fetal growth trajectory size do appear to influence the risk for childhood asthma symptoms; for both cohorts reduced early growth was associated with increased asthma symptoms (Table 2). In later pregnancy, the Aberdeen group report increased asthma associated with increased growth in late pregnancy but in contrast, the Southampton group observed growth failure was associated with increased symptoms (Table 3). Second, early growth acceleration was associated with increased risk for atopy and atopic conditions in both cohorts; interestingly this pattern is the opposite predicted by the Barker hypothesis [10] but this was proposed for cardiovascular outcomes and not atopy. Other cohorts in Australia and Netherlands can shortly be expected to report on asthma and associated outcomes in the context of fetal growth and these will be welcome additions to the present literature. What is clear is that factors which influence fetal size and growth are important to childhood asthma outcomes and antenatal interventions may prevent childhood asthma, for example, by influencing maternal diet or smoking.

## Figures and Tables

**Table 1:** Summary of studies linking birth weight to asthma.

Study reference	Year of birth	Country	Asthma Outcome	Age at follow up	Number in cohort	Positive or negative	Magnitude of effect*
[21]	1928–1952	Sweden	Doctor diagnosed asthma, asthma admission or death	36–70 years	21,588 twins	Negative	OR for 2 kg 1.58 [1.06, 2.38] compared to 2.5 kg
[27]	1947–1973	Nordic-Baltic countries	Wheeze, wheeze with shortness of breath	20–47 years	1683	No association	Wheeze reduced by 2% [±19%] for each 500 g wt gainBirth weight 2500 versus 4000 g linked with 8% increase in FEV1
[32]	1966	Finland	Doctor diagnosed asthma ever and symptoms in last 12 months	31 years	4719	No association	Using ponderal index tertiles and middle as reference, risk for asthma in lowest 1.14 [0.78, 1.65] and for highest 1.22 [0.85, 1.75]. Ponderal index had significant U-shaped relationship with skin prick positivity
[15]	1970–1989	UK	Hospital admission for asthma	2–10 years	248612 recruited 4017 admitted	Negative	Risk increased 20% [10–30] comparing 1–3 kg versus 3–4 kg
[30]	1975–1979	Finland	Life time prevalence doctor-diagnosed asthma	16 years	3065 twin pairs	No association	OR 0.61 [0.30, 1.24] for 2.5–3 kg versus <2 kg. OR highest versus lowest quartile for ponderal index (wt/length^3^) 1.82 [1.18, 2.79]
[26]	1975–1988	UK	Asthma diagnosis	13–14 years	10,809	No association for birth weight	Highest versus lowest quintile head circumference increased hay fever (1.23 [1.03, 1.47]). Highest quintile birth wt increased hayfever (1.17 [0.99, 1.39]). Highest versus lowest birth weight 0.92 [0.62, 1.35]
[25]	1977–1980	Australia	Asthma	Mean 14 years	180 preterm and 42 term deliveries	No association	Asthma prevalence 21% in controls, 21% in 1–1.5 kg birth wt and 15% in 0.5–1 kg birth wt
[35]	1984–1987	Denmark	Hospital admission for “definite” or “any” asthma	12 years	10440	Positive	Definite asthma increased 1.62 [1.02, 2.59] for above compared with below average birth weight. More convincing relationship between increasing ponderal index and any and definite asthma admission
[34]	1985–1988	Canada	Emergency visits for asthma	10 years	83,595 children	Positive above 4.5 kg	Above 4.5 kg increased risk (1.16 [1.04, 1.29]) compared to normal weight. Beyond 4.5 kg 10% increase risk [2, 19].
[33]	1986	Finland	Doctor diagnosed asthma	16	9479	Positive at very highest weight	Highest birth wt (>4.51 kg) had greatest atopic asthma risk 2.4 [1.33, 4.32] compared to 2.5–3.34 kg
[16]	1987	Finland	Hospitalisation or free entitlement to asthma medication	7 years	60254	Negative	Birth wt < 2.5 kg OR for asthma 1.83 [1.50, 2.24] independent of maternal smoking
[14]	1988	USA	Physician diagnosed asthma by age 3 years	0–4 years	8071	Negative	<1.5 kg OR 2.9 [2.3, 3.6], 1.5–2.5 kg OR 1.4 [1.1, 1.8] compared to ≥2.5 kg
[23]	1988–1990	Netherlands	Parent reported asthma	Mean 6 years	1961	No association for birth weight	Relationship between asthma and gestational age (risk for >36 weeks 2.0 [1.0, 4.0] compared to 40 weeks) and asthma and head circumference : birth weight ratio (risk for above median 1.8 [1.1, 3.2] compared with below median).
[20]	1992–1998	Sweden	Ever had asthma	9–12 years	446 twins	Negative	OR 1.57 [1.38, 1.79] for each kg decrease
[28]	1994–1996	Sweden	Wheeze	4 years	2869	No association for birth weight	Birth length ≥ 90th centile OR any wheeze 0.4 [0.21, 0.77]
[29]	1994–1996	USA	Physician diagnosed plus wheeze in the last year	6 years	454 at risk for asthma	No association	Birth weight < 2.5 kg OR asthma 1.05 [0.40, 2.73]. Gestation < 38.5 weeks assoc with increased asthma (OR 4.7 [2.1, 10.5])
[17]	1994–2000	Denmark	History of asthma	3-9 years	8280 twin pairs	Negative	Asthma assoc with 122 g lower birth weight [85, 160]. Risk increased by 4% per 100 g wt reduction
[31]	1995–2001	Canada	Hospital admission or >1 physician visits with asthma over 2 years	6	687,194	No association	Extremely heavy (>6.5 kg) OR 1.21 [0.67, 2.19]
[22]	Approx 1995–2001	USA	?	1–5 years	2410	Negative	Linear 20% increase risk [2, 35] for each kg reduction in birth weight. Breast feeding apparently protective of influence of low birth weight
[24]	1996-1997	Netherlands	Doctor diagnosed	Mean 7 years	3628	No association	Relationship between birth weight and wheeze (risk increased by 17% [1, 35] for each kg reduction in birth weight
[18]	1996–2004	Finland	Asthma diagnosis and prescribed inhaled steroids or montelukast	Three years	20,623 case-control pairs	Negative	Birth weight < 2.5 kg OR 1.40 [1.20, 1.60]
[19]	1998–2000	USA	Asthma diagnosis	3	1803	Negative	Birth weight < 2.5 kg OR 2.36 [CI not given]

* OR=odds ratio for asthma. Numbers in square brackets correspond to 95% confidence intervals.

**Table 2:** Summary of asthma and allergy outcomes in the context of changing growth trajectory during early pregnancy.

First-second trimester	Increased rate of growth			Reduced rate of growth		
	Asthma symptoms	Atopy	Lung function	Asthma symptoms	Atopy	Lung function

Southampton 3 years		Increased skin prick positivity		Increased nonatopic wheeze		
Aberdeen 5 years	Increased asthma		Reduced FEV_1_, FVC, FEF_25–75_			Reduced FEV_1_
Aberdeen 10 years	Increased asthma	Increased hayfever	Reduced FEF_25–75_	Increased asthma	Reduced eczema	Reduced FVC

**Table 3:** Summary of asthma and allergy outcomes in the context of changing growth trajectory during late pregnancy.

Second-third trimester	Increased rate of growth			Reduced rate of growth		
	Symptoms	Atopy	Lung function	Symptoms	Atopy	Lung function

Southampton				Increased atopic wheeze	Increased skin prick positivity	
Aberdeen 5 years	Increased asthma*					
Aberdeen 10 years	Increased asthma		Reduced FEV_1_ and FVC			

*Data not published but can be confirmed by the author.
